# Supplementary material for: Comparison of Resistance Spectra after First and Second Line Osimertinib Treatment Detected by Liquid Biopsy
Source: Cancers (Basel). 2021 Jun 8;13(12):2861. doi: 10.3390/cancers13122861 (PMC8227553; doi:10.3390/cancers13122861)
Supplement: Supplementary file 1 [file cancers-13-02861-s001.zip › cancers-1214406-supplementary.pdf]

cancers

MDPI

Supplementary

Comparison of Resistance Spectra After First and Second Line Osimertinib Treatment Detected by Liquid Biopsy

Balázs Jóri, Stefanie Schatz, Len Kaller, Bettina Kah, Julia Roeper, Hayat O. Ramdani, Linda Diehl, Petra Hoffknecht, Christian Grohé, Frank Griesinger, Markus Tiemann, Lukas C. Heukamp and Markus Falk

Table S1. Patient treatment and alterations detected with liquid biopsy in the 56 patient's cohort of late stage NSCLC during disease progression under TKI treatment. (X) mutation present from a single nucleotide change; (XX) mutation present from two nucleotide changes leading to C797S: (c.2389T>A and c.2390G>C) ; *TP53* in bold letter because of *RB1* mutations suggesting a small-cell transformation SCLC. Abbreviations: erlotinib (ERL), gefitinib (GEF), rociletinib (ROC), afatinib (AFA), osimertinib (OSI). Varaiant of unknown significance (VUS).

			***EGFR* dependent resistance**			***EGFR* Independent Putative Resistance**					**Passenger Mutation**	
patient	*EGFR* driver confirmed by liquid biopsy	TKI received	T790M	C797S	other	amplifica-tion	SCLC/SCLC switch	kinase switch	fusion	alternate pathway	(in)activating	VUS
P15	E746_A750del	OSI		X							*TP53* (R110L)	*PIK3CA* (K111del)
P55	L858R	OSI			T854A	*MET*			*ALK-EML4* (v3)		*TP53* (R248Q)	*MET* (A179T)
P29	L858R	OSI					*RB1* (R787*)				*STK11* (L105*); ***TP53*** (G244V)	
P27	L858R	OSI									*TP53* (E336fs*10)	*KEAP1* (E134K)
P43	L858R	OSI									*BRCA2* (E2981fs); *TP53* (Y234C)	*ALK* (R1084H); *PDGFRA* (R829*)
P46	L861Q	OSI										*BRCA1* (I562V)
P49	E709G, G719S	OSI									*TP53* (R156P, E298*)	*TSC2* (I723V); *ATM* (E586_G587delinsDC, A425V); *FGFR3* (E330Q); *CTNNB1* (K435N)
P20	*non*	OSI										
P30	*non*	OSI										
P44	E746_S752delinsV	ERL, OSI	loss of		G724S							*ATM* (K3004E)
P16	E746_S752delinsV	AFA, OSI, CRI	loss of			*EGFR*	*RB1* fusion (intergenic)				***TP53*** (Q167*); *STK11-STK11*	*RB1* (T502I); *CTNNB1* (I231M); *TSC2* (D1406N)
P51	E746_A750del	AFA, OSI	loss of			*MET*					*TP53* (R175H)	*ROS1* (R448H)
P14	L858R	ERL, OSI	loss of			*MET*						*FGFR1* (R749H)
P32	E746_A750del	AFA, OSI	loss of					*ERBB2* (V777L, I767M, S310Y)		*KRAS* (G13D)	*BRCA2* (T3030fs, N2134fs); *TP53* (R249S)	

P37	E746_A750del	ERL, OSI	loss of						*RET-RUFY1*	*PIK3CA* (E545K, E542K)	*TP53* (Q136P)	*EGFR* (R932C); *PIK3CA* (R87T)
P06	E746_A750del	AFA, OSI	loss of							*KRAS* (G12D)	*ATR* (R2148*); *TP53* (W91*)	*ATR* (H1520R); *MTOR* (D2512Y, R2505*); *RET* (A641T)
P42	E746_A750del	AFA, OSI	loss of							*PIK3CA* (E545K)	*CDKN2A* fusion (intergenic)	*ERBB2* (T216M)
P41	E746_A750del	AFA, OSI	loss of								*TP53* (Q192*)	
P19	L747_S752del	ERL, AFA, OSI	loss of									*MTOR* (M2366V)
P04	K745_A750delinsK	ERL, OSI	loss of								*TP53* (R158P)	
P17	L747_T751delinsP	GEF, AFA, OSI	loss of									*PDGFRA* (E336G)
P11	E746_A750del	GEF, OSI	loss of									
P38	E746_A750del	GEF, OSI	loss of									*ALK* (P230Q), *TSC2* (R905Q)
P35	L858R	AFA, OSI	X	X	L718V, L718Q, L792H, G796S	*EGFR*					*TP53* (*c.376-2A>T*)	*FGFR3* (D760N); *TSC2* (R1451S); *BRCA1* (K556N)
P31	L747_P753delinsS	GEF, ERL, AFA, OSI	X	X		*EGFR*						
P08	L858R	ERL, OSI	X	X			*RB1* (G203fs)				***TP53*** (E198*)	
P12	L858R	ERL, OSI	X	X				*ERBB2* (S310F)				*ALK* (G35fs)
P23	L858R	AFA, OSI	X	X					*AGK-BRAF*	*CTNNB1* (S37Y)		
P56	L747_P753delinsS	AFA, OSI	X	X					*DLG1-BRAF*		*IDH2* (R140Q); *TP53* (Q104*, S241F)	*EGFR* (M1002T)
P13	E746_T751delinsA	AFA, OSI	X	X						*CTNNB1* (S37Y)	*TP53* (G199V)	
P33	E746_A750del	AFA, OSI	X	X						*MTOR* (Y1974H)		*ATM* (H1352Q)
P24	L747_T751delinsP	AFA, OSI	X	X							*TP53* (R248Q)	
P02	K745_A750delinsK	AFA, ROC, OSI	X	X								*TSC1* (A659V)
P18	E746_A750del	ERL, OSI	X	X							*TP53* (V157F)	*EGFR* (G459A)
P39	E746_A750del	GEF, OSI	X	X							*CTNNB1* (S37Y)	*ATM* (N1719D)
P03	K745_A750delinsK	ERL, OSI	X	XX	V843I	*EGFR*				*KRAS* (A146V)		*DDR2* (S215F), *ROS1* (I595del)
P05	E746_A750del	ERL, ROC, OSI	X	XX					*TACC-FGFR3*			*TSC1* (R992T)
P48	E746_A750del	AFA, OSI	X	XX							*TP53* (R158L)	
P28	E746_A750del	AFA, OSI	X	XX								*ARAF* (G499E), *MET* (M1247T), *TP53* (I162N)
P36	L858R	ERL, OSI	X		L718Q	*MET*	*RB1*				***TP53*** (V73fs)	

					(S114fs, *c.1960+1G>A*)				
P54	L858R	AFA, OSI	X	L792H		*RET* (V804L)		*TP53* (I254N)	
P07	L858R	AFA, OSI	X						
P22	E746_A750del	AFA, OSI	X					*ATM* (*c.2921+1G>T*)	*ATR* (S2264L), *NFE2L2* (L266F), *EGFR* (R962C)
P34	E746_A750del	AFA, OSI	X					*TP53* (R248Q)	
P01	L747_P753delinsQ	ERL, OSI	X					*ATM* (R3008H)	
P21	L858R	GEF, OSI, AFA	X					*TP53* (V216L)	*ERBB2* (D1252N)
P10	E746_A750del	AFA, GEF, ERL, OSI					*PTEN* (S399fs)	*TP53* (H179Y)	*MTOR* (R731H)
P52	E746_A750del	AFA, GEF, OSI						*TP53* (R249G)	
P26	E746_A750del	AFA, OSI							
P45	E709V, G719C	ERL, OSI						*TP53* (I195N,W53*)	
P53	*non*	AFA, GEF, OSI							
P09	*non*	AFA, OSI							
P47	*non*	AFA, OSI							
P50	*non*	AFA, OSI							
P40	*non*	ERL, OSI							
P25	*non*	GEF, OSI							
